# Preparation and physicochemical characterization of whitlockite/PVA/Gelatin composite for bone tissue regeneration

**DOI:** 10.3389/fchem.2024.1355545

**Published:** 2024-02-14

**Authors:** Sadaf Batool, Usman Liaqat, Zakir Hussain

**Affiliations:** School of Chemical and Materials Engineering (SCME), National University of Sciences and Technology (NUST), Islamabad, Pakistan

**Keywords:** whitlockite, bone mineral, calcium phosphate, tissue regeneration, composites

## Abstract

This work used a straightforward solvent casting approach to synthesize bone whitlockite (WH) based PVA/Gelatin composites. WH nanoparticles (NPs) were synthesized using the wet precipitation method, followed by their addition into the PVA/Gelatin matrix at concentrations from 1% to 10%. The physicochemical characterization of the prepared PVA/Gelatin/WH composite was carried out using ATR-FTIR, Optical profilometry, a Goniometer, a Universal tensile testing machine (UTM), and scanning electron microscopy (SEM) techniques. The ATR-FTIR analysis confirmed the formation of noncovalent interactions between polymeric chains and WH NPs and the incorporation of WH NPs into the polymer cavities. SEM analysis demonstrated increased surface roughness with the addition of WH NPs, supporting the results obtained through optical profilometry analysis. The mechanical properties of the prepared composite showed an increase in the tensile strength with the addition of WH filler up to 7% loading. The prepared composite has demonstrated an excellent swelling ability and surface wettability. The reported results demonstrate the exceptional potential of the prepared composite for bone tissue regeneration.

## 1 Introduction

Conventional autologous and allogeneic bone grafts have several clinical limitations, and they are now replaced by synthetic alternatives that have shown promising results for bone tissue regeneration over the past 2 decades. The most reliable synthetic approach developed to date is the calcium phosphates (CaPs) based polymeric composites (Sharma et al., 2016). This ceramic polymer combination emulates a natural extracellular matrix (ECM) where the ceramic phase gives rigidity and mechanical properties while the polymeric phase provides elasticity, non-toxicity, and biocompatibility (Govindan et al., 2020).

Calcium phosphates like hydroxyapatite (HA) (Sattary et al., 2019), monetite (Matinfar et al., 2019), Tricalcium phosphate (α and β-TCP) (Baheiraei et al., 2017; Lu et al., 2021), and brushite (Cabrejos-Azama et al., 2014) have been explored extensively for bone tissue regeneration applications. However, their poor mechanical strength, uncontrolled degradation, less stability under the physiological environment, and bioinert nature have limited their use for the clinical application of bone tissue regeneration (Kumta et al., 2005; Kaur et al., 2014; Goldberg et al., 2015; Habraken et al., 2016). To improve these properties, scientists are now working to incorporate different essential metals like magnesium (Mg) (Craciunescu et al., 2011; Ramesh et al., 2016), strontium (Sr) (Ge et al., 2018), sodium (Na) (Gunawidjaja et al., 2010), and zinc (Zn) (Zheng et al., 2016) in the CaPs. These composites have enhanced biological response but are amorphous and have poor stability (Ramesh et al., 2016; Nouri-Felekori et al., 2019). To further improve these properties polymeric composites (Rezwan et al., 2006; Chung et al., 2016; Głab et al., 2021) and coatings (Ur Rehman et al., 2017; Dhinasekaran et al., 2021; Ritwik et al., 2022) of CaPs are also fabricated. However, a recent analysis of native bone has suggested that there is another magnesium-containing phase present in bone during the earliest stages of bone growth. This phase was identified as whitlockite (Lagier and Baud, 2003; Magalhães and Costa, 2018; Muthiah Pillai et al., 2019a; Kim et al., 2019).

Whitlockite (Ca_18_Mg_2_(HPO_4_)_2_(PO_4_)_12_, the second most abundant phase of bone CaPs, has good biological properties, negative surface charge, mechanical strength, stability in the body’s physiological environment, and is bioresorbable. It has magnesium that plays a critical role in pH regulation of physiological fluids and supports bone growth during earlier stages of its development (Muthiah Pillai et al., 2019b; Batool et al., 2020). This all makes it an ideal material for bone tissue regeneration application. Previously, Bauer et al., 2021 reported good compression strength and a good expression of collagen type I of WH. It has been found to show excellent potential to deposit calcium ions on its surface. In another work, Muthiah Pillai et al., 2019b incorporated WH into the collagen matrix to synthesize injectable hydrogels that were cytocompatible and showed good prevention from hemostasis. Similarly, Jin et al., 2019 have studied WH for bone healing in the Ilium defect Rabbit model and reported higher ALP activity, cell proliferation, and an increased bone volume.

Furthermore, Kim et al., 2017 did *in situ* modeling of WH NPs and confirmed the critical role of WH during earlier stages of bone development. All these studies have demonstrated the excellent potential of WH for bone tissue regeneration. The present study reports the preparation of the PVA/Gelatin composite, its characterization, and physiochemical analysis. Gelatin and PVA have been combined previously in different studies because of their excellent mechanical strength and bioactivity and have been evaluated for various biological applications like wound dressings, tissue regeneration, and drug delivery (Wang et al., 2008; Linh et al., 2010; Raafat and Abd-Allah, 2018). Composites of WH with CaPs have also been prepared to improve their mechanical strength (Nayar and Sinha, 2004; Wang et al., 2010; Basak et al., 2018) and to evaluate their potential for bone tissue regeneration applications (Yang et al., 2010; Ghorbani et al., 2016; Lian et al., 2018; Xue et al., 2018). Gelatin is an acid-hydrolyzed, denatured triple-helical polypeptide collagen complex with low antigenicity. It contains the RGD (Arg-Gly-Asp) factor that is important for cell adhesion in the natural system (Dasgupta et al., 2019; Kenawy et al., 2019; Govindan et al., 2020). Similarly, PVA is a hydrophilic, biocompatible, crystalline, non-toxic, economical synthetic polymer with good mechanical properties and physicochemical stability (Jayasekara et al., 2004; Głab et al., 2021). In the present study, a PVA/Gelatin blend has been prepared with different ratios keeping gelatin as the significant component of the matrix. Our aim was to prepare a blend with good mechanical strength as well as good physicochemical properties that could be used for bone tissue regeneration application. The mixture was evaluated for its mechanical strength from 1 wt% to 10 wt%, and their mechanical strength was measured in triplicate. The results indicated that a polymeric blend with 8 wt% gelatin and 2 wt% PVA has excellent mechanical strength. The optimized composition was selected to add WH NPS as filler in different concentrations to study their effect on the physicochemical and biological properties of the PVA/Gelatin blend. WH NPs were prepared using the precipitation method reported earlier by us.

## 2 Materials and methods

Polyvinyl alcohol (fully hydrolyzed) was purchased from Merck (Germany), Calcium hydroxide (≥96%) from GPR Rectapur, Orthophosphoric acid (98 g/mol) from Honeywell (Fluka, NC, United States), Gelatin (Type A), and Magnesium hydroxide (95%) from Duksan (Daejeon, South Korea). All chemicals were used as received without any further purification.

### 2.1 Synthesis of whitlockite

Whitlockite was prepared using the wet precipitation method reported previously (Batool et al., 2020). Briefly, 0.13 M solution of Mg (OH)_2_ and 0.37 M Ca(OH)_2_ was prepared, mixed, and stirred for 20 min at 50°C. Following the process, 0.5 M H_3_PO_4_ was added to the above mixture at 10 mL/5 min under stirring to achieve a pH of 5. The whole mixture was then heated at 100°C for another 10 h with subsequent ageing at room temperature for 14 h. The precipitates were filtered, washed thrice with deionized water, and dried overnight at 50°C to obtain WH NPs.

### 2.2 Synthesis of Gel/PVA blend

The solution casting method was used to prepare gelatin and PVA blend. Hence, 2 wt% PVA solution was prepared in deionized water by stirring at 80°C for 3 h and 8 wt% gelatin solution was prepared in deionized water by mixing at room temperature for 30 min. Both mixtures were mixed at room temperature and stirred for another 4 h followed by the addition of this mixture into a plastic petri plate and air dried overnight.

### 2.3 Synthesis of Gel/PVA/WH composite

The above-mentioned procedure was used for the preparation of Gel/PVA/WH composite, except that the WH NPs were sonicated in deionized water for 1 h before being added into the polymer blend at step two where PVA and gelatin mixtures were mixed followed by stirring the mixture for 4 h before casting. WH NPs were loaded in a PVA/gel matrix from 1 wt% to 10 wt%.

## 3 Characterization

### 3.1 Chemical characterization

The prepared WH NPs and PVA/Gel/WH composite were characterized using different techniques. Phase analysis of the prepared WH NPs was carried out using the DRON-8 Bourevestnik (Saint-Petersburg, Russia) X-ray diffraction machine. To confirm the functional groups of WH NPs, Raman spectroscopic analysis was carried out using BWS415-532S-Raman manufactured by BW TEK INC (Newark, NJ, United States) and Fourier Transform Infrared Spectroscopy (FTIR) by PerkinElmer, spectrumTM100 spectrophotometer. ALPHA platinum ATR by Bruker was used to confirm the functional groups of the PVA/Gel/WH composite. For the surface analysis, scanning electron microscopy (SEM) by JEOL JSM-64900 was used.

### 3.2 Physical characterization

The mechanical strength of the PVA/Gel/WH composite was measured using a universal testing machine (Shimadzu AGX Plus, Kyoto, Japan) according to the ASTM D882 standard. Specimens with 2 cm × 1 cm × 0.1 mm (L × W × T) dimensions were prepared for the analysis, and a 20 kN load was applied at 1 mm/min constant crosshead speed. The data were obtained by averaging three samples of each composition.

For the wettability test, the Kruss DSA 25 (Hamburg, Germany) Goniometer was used to determine the surface hydrophilicity of the prepared composite films. Specimens with 2 cm × 2 cm were prepared from each composition, and a sessile drop of water was placed on the model at room temperature. The surface hydrophilicity was measured by averaging three readings from each sample.

To measure the surface roughness of the prepared samples, A 2D non-contact optical profilometer (Nanovea, United States, Model PS50) was used where 1.0–1.5 cm long specimens were prepared for the test.

Similarly, for the swelling test, the conventional gravimetric method was used to measure the prepared composite’s swelling ability. In this case, 1 × 1 cm specimens were cut from each composition, weighed (two), and immersed in PBS solution (pH 7.4) at 37°C for 24 h. After 24 h, the samples were removed from the PBS solution, pressed gently with tissue paper to remove surface water, and weighed (wt). The percentage swelling was calculated by using the following formula.

$$Degree\;of\;Swelling=\frac{W_{t-}W_{o}}{W_{o}}\;x\;100$$

The swelling percentage was measured by averaging three readings from each sample, and the results were reported as mean and standard deviation.

For *in vitro* degradation test, the prepared composite was studied using ASTMF 1635–95 standard. In this case, 1 × 1 cm specimen of each blend was immersed in PBS solution at 37°C for 30 days. The initial weight of the specimen before immersion was denoted as Wo and the final weight as Wt. The degradation behavior was measured after 0, 5, 10, 15, 20, 25, and 30 days. After a specific time, the sample was removed from PBS media, dried at 37°C for 48 h, and weighed.

## 4 Results and discussion

### 4.1 Characterization of WH NPs

**FIGURE 1:**
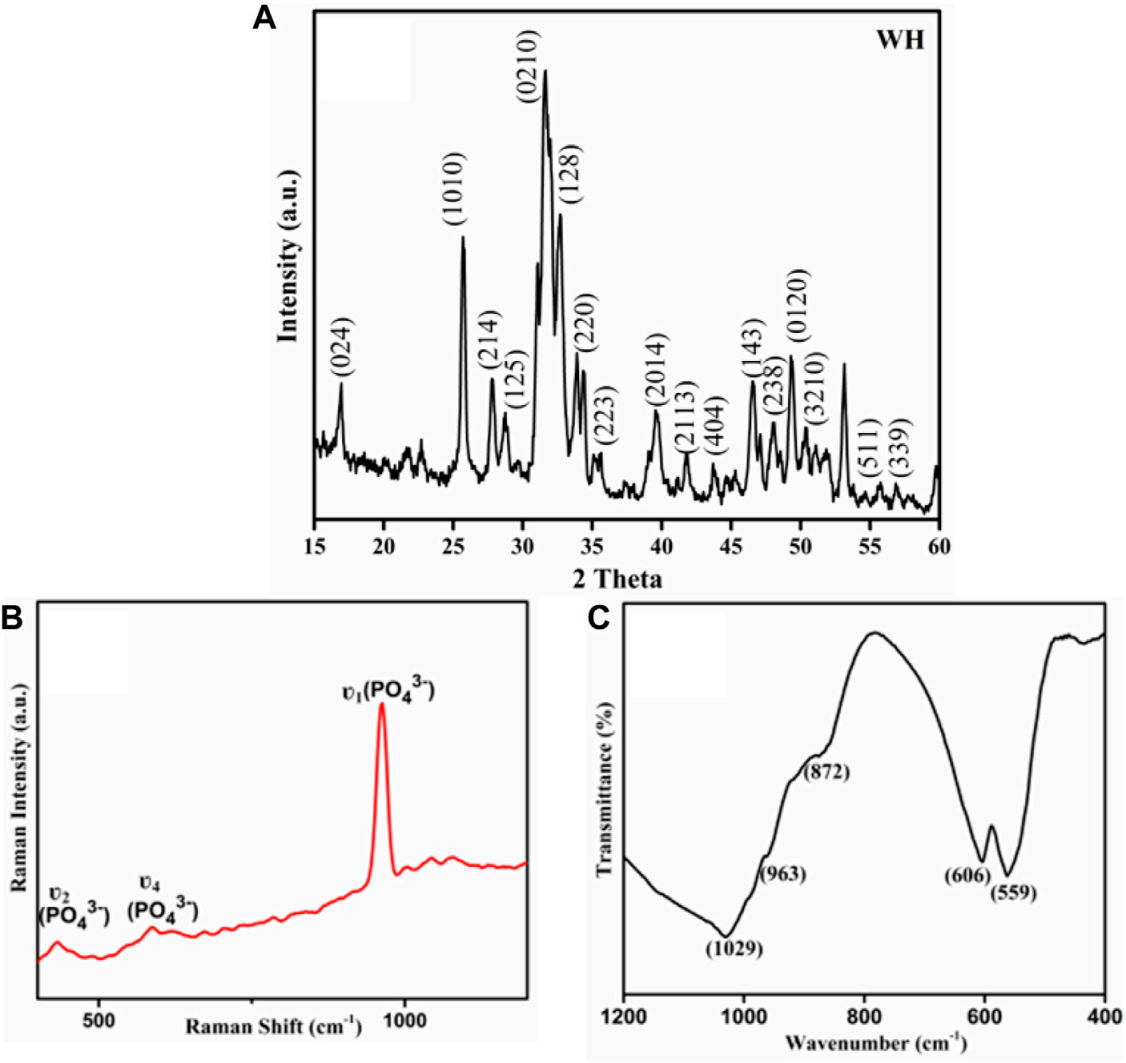
**(A)** XRD spectrum of WH NPs **(B)** Raman of WH NPs **(C)** FTIR of WH NPs.

The x-ray diffractogram showed the characteristic peak for WH (Figure 1A) with hkl (1010), (024), (214), (128), (0210) matched exactly with the JCPDS (01-070-2064 and 00-042-0578) and literature (Guo et al., 2018; Hu et al., 2019; Jin et al., 2019; Batool et al., 2020; Lee et al., 2020; Wang et al., 2020). The Raman spectrum of WH NPs (Figure 1B) showed the characteristic ʋ1 and ʋ4 vibrations of the $PO_{4}^{3-}$ group at 962 cm^−1^ and 586 cm^−1^. The peak at 432 cm^−1^ is the characteristic of ʋ2 vibrations of the $PO_{4}^{3-}$ group (Khan et al., 2013; Hu et al., 2019; Batool et al., 2020). The formation of WH NPs was further supported by the FTIR spectroscopic analysis. The FTIR spectrum of the prepared WH NPs (Figure 1C) showed the characteristic peak of $HPO_{4}^{2-}$ at 872 cm^−1^. The stretching and bending vibrations of the $PO_{4}^{3-}$ group appeared at 1029 cm^−1^, 963 cm^−1^, 606 cm^−1^, and 1120 cm^−1^ in the given spectrum which are the characteristic peaks of CaPs. The peak at 872 cm^−1^ is the characteristic peak of WH. The FTIR data suggested the phase purity since peaks at 650 cm^−1^ and 3570 cm^−1^, characteristic of the secondary CaP phase are absent in the present case (Kim et al., 2017; Yang et al., 2020).

### 4.2 Characterization of PVA/Gelatin/WH nanocomposite

**FIGURE 2:**
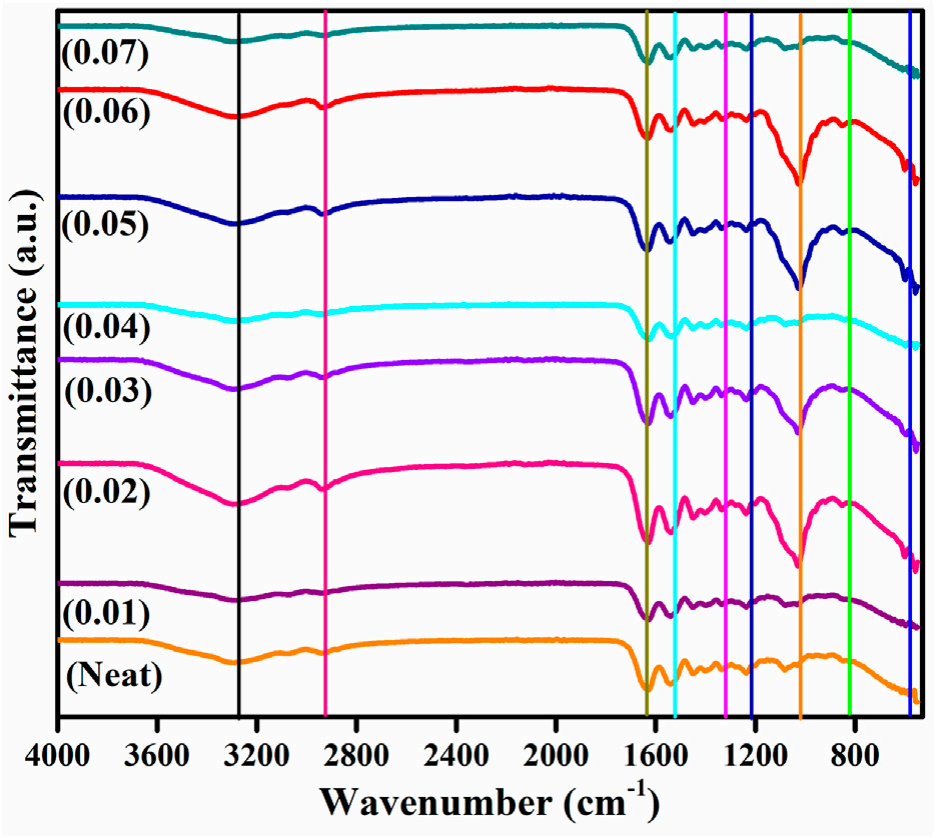
ATR Spectrum of PVA/Gel/WH Composite with different composition, neat film (orange), 0.01 WH (magenta), 0.02 (pink), 0.03 (blue), 0.04 (turquoise), 0.05 (royal blue), 0.06 (red), 0.07 (zinc) of WH NPs.

Figure 2 shows the attenuated total reflectance spectrum of the prepared nanocomposite. The broad peak at 3283 cm^−1^ is the characteristic stretching vibration of the hydroxyl group of PVA, confirming the presence of alcoholic terminal groups. A peak at 2,922 cm^−1^ is the stretching vibrations of C-H interconnections of the PVA backbone, while a peak of 1326 cm^−1^ is characteristic of the CH_2_ bending vibrations of the PVA backbone. Also, a peak at 1238 cm^−1^ could be associated with C-O bending vibration of the alkyl group of PVA or C-N stretching of the amine group of porcine-derived gelatin, confirming the presence of Amide III (Tedesco et al., 2016). The peak at 843 cm^−1^ is the C-O stretching vibration of PVA (Jayasekara et al., 2004). The characteristic peaks at 1641 and 1535 cm^−1^ are the distinct peaks for amide I and II functionalities of the gelatin. The presence of C=O bond stretching vibrations confirms the dominance of alpha-helix in protein conformation. At the same time, the N=H bond couples with C≡N and generates bending vibrations of amide II. Since newly formed bands could not be observed, the prepared blend was assumed to be linked physically as a separate phase (Treesuppharat et al., 2017; Anugrah et al., 2020; Liu et al., 2020; Yadav et al., 2020). The peaks at 601 cm^-1^ and 1081 cm^-1^ establish the presence of the $PO_{4}^{3-}$ functional group, confirming the embedding of filler within the polymer matrix (Uma Maheshwari et al., 2014). However, the phosphate ion peak in the composite appeared slightly in higher concentrations, which could be due to the dominance of peaks of solid polymer functional groups.

**FIGURE 3:**
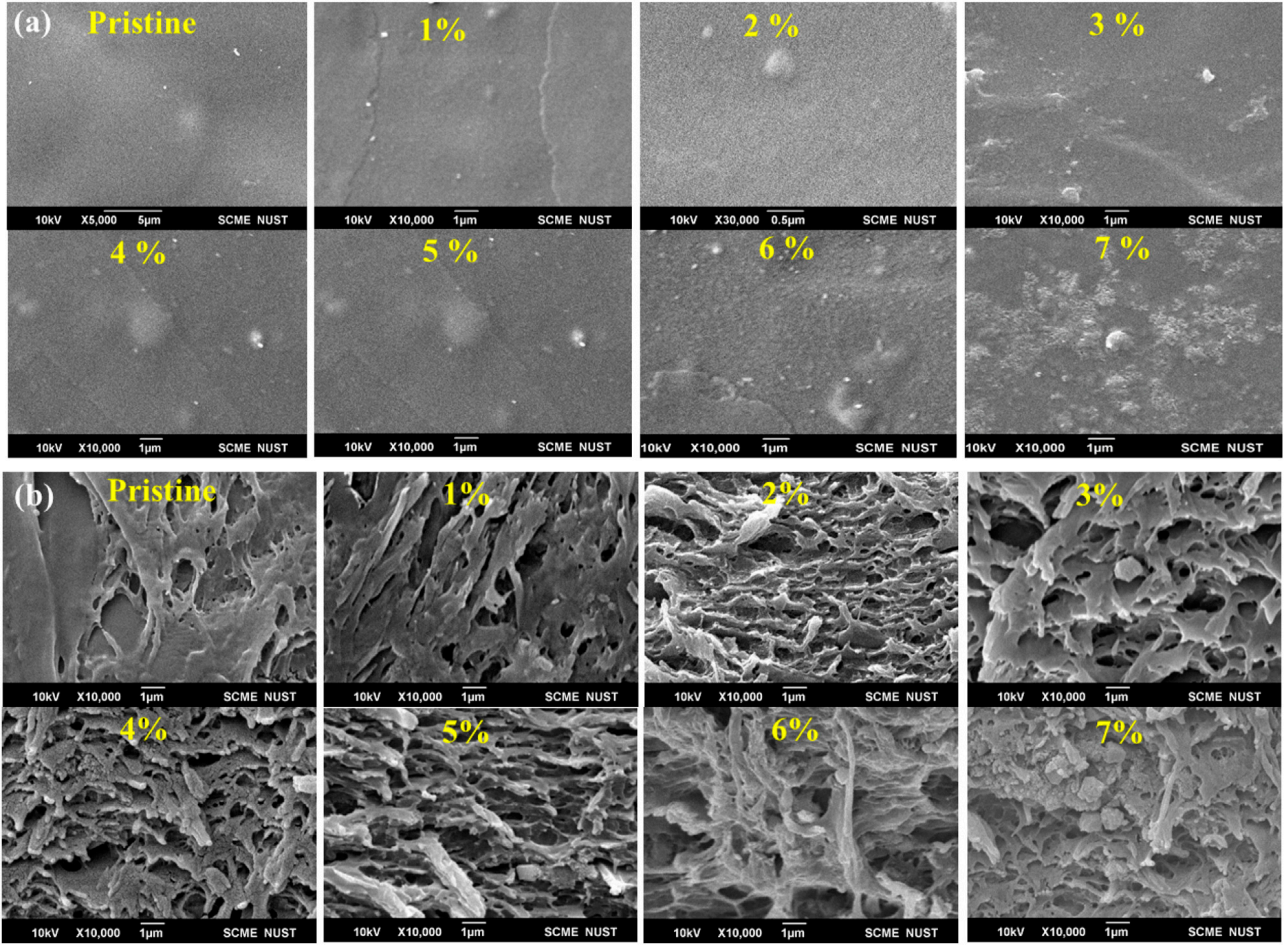
**(A)** SEM images of the Surface of PVA/Gelatin/WH Composite **(B)** Cross-sectional images of PVA/Gelatin/WH Composite.

The SEM images of the pristine blend represent a smooth surface whereas surface roughness increased by increasing the concentration of WH from 1 wt% to 7 wt% in the polymer blend. Such observations are in close agreement with the reported literature. In the present case, pure PVA/Gelatin blend has a smooth surface, and the addition of CaPs increased surface roughness, suggesting that at lower concentrations, the filler is distributed evenly into the polymer matrix, which is not the case at higher concentrations due to agglomeration (Figure 3A). The cross-section images of the composite show a dense surface of the PVA/Gelatin blend while a porous surface could be observed due to the addition of filler (Figure 3B). This porous network is ideal for bone tissue regeneration application as it allows vascularization as well as facilitates the movement of fluid in and out of the scaffold. At higher concertation, the WH NPs are also visible in the cross-section, confirming the successful incorporation of NPs into the polymer matrix (Tedesco et al., 2016; Azizi-Lalabadi et al., 2020).

### 4.3 Mechanical properties of the prepared PVA/Gelatin/WH composites

It has been reported earlier that gelatin has an excellent tensile strength (17.51 MPa) but poor elongation at break (7.86%) (Yang et al., 2010; Tedesco et al., 2016). The mechanical strength of gelatin films comes from the triple-helical complex. The adjacent polymer interactions in gelatin are responsible for the highest strength as gelatin is a protein and is rich in non-ionized polar amino acids, forming hydrogen bonds to add high cohesion and low flexibility (Tedesco et al., 2016). Also, PVA has an excellent tensile strength (22.31 MPa) and elongation at break (10.96%), combination of both results in an increase in the tensile strength (37.63 MPa). This increase in tensile strength suggests noncovalent intermolecular interactions between PVA and gelatin chains during the formation (Yang et al., 2010). The elongation of PVA films comes from the straight-chain structure and its more robust backbone. The toughness of PVA can cover the brittle nature of gelatin. The ideal scaffold must have the required mechanical strength to sustain the pressure during healing and not be crushed during the implantation. The PVA can compensate for the rigid association of gelatin functionalities. While gelatin does not impart a specific increase in mechanical strength, its RGD factor and suitable wettability properties are essential for promoting the cellular responses of the scaffolds (Ghorbani et al., 2016).

Since PVA is hydrolytic; its concentration above a specific limit cannot be increased. PVA and gelatin form inter/intra chains hydrogen bonds that can prevent the sudden loss of mechanical strength in a physiological environment. A sudden weight change may appear after implantation, but it can become constant after some time as PVA and gelatin can form noncovalent interactions with themselves and with the surrounding media (Linh et al., 2010). The strength of PVA/Gelatin can also be increased by adding hydrophilic filler in the polymer matrix that could easily fit into the protein chain of gelatin and make polar interactions with PVA side chains. The filler also prevents protein and PVA side-chain self-interactions by establishing new noncovalent interactions with it and improving the strength of the polymeric blend. A study on HA containing PVA/Gelatin has demonstrated an increase in mechanical strength with the addition of HA (Chaudhuri et al., 2016; Meshram et al., 2017). Such observations have also been reported for a composite of WH with PLLA and PCL where the mechanical strength of the composite increased to 2.81 MPa with 10% loading of the (Januariyasa et al., 2020; Zhang et al., 2021).

**FIGURE 4:**
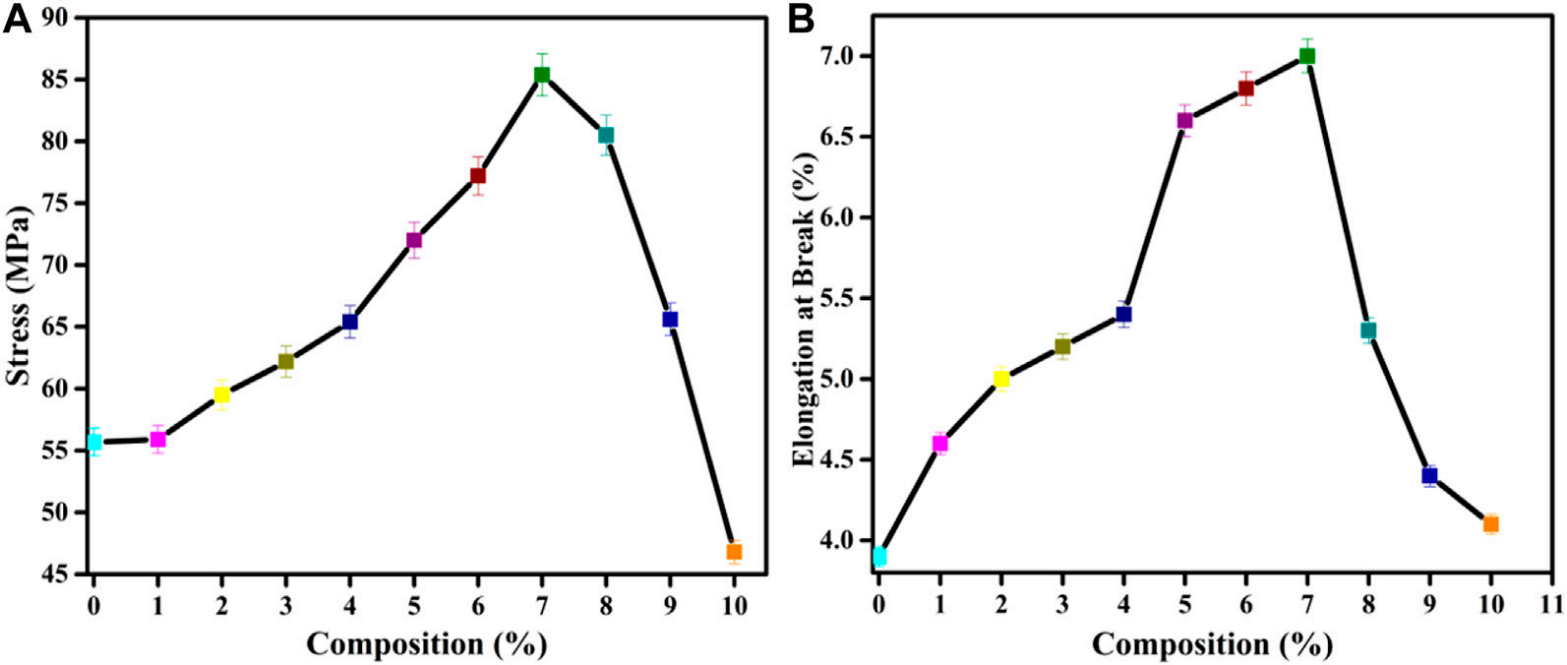
**(A)**Tensile Strength of the PVA/Gelatin/WH Composites **(B)** Elongation at break of PVA/Gelatin/WH Composite.

In the present investigation, the maximum tensile strength obtained is at 7 wt% loading of the WH, which is far higher than the PCL/WH composite and 153% more than the pristine polymer blend (Figure 4A). Similarly, strain increased linearly with the addition of WH up to 7 wt% loading, and then a gradual decrease in pressure was observed till 10 wt% loading of the WH (Figure 4B). The increase in the strain was 179% as compared to the pristine blend. The tensile strength of the polymeric composite depends upon the physicochemical interactions of filler and polymeric combination, its dispersion in a polymer matrix as well as concentration. An increase in the elongation at break and tensile strength after adding WH confirms the nanoparticles’ reinforcing effect.

### 4.4 *In-vitro* degradation of PVA/Gelatin/WH composites

The degradation studies were conducted to determine how a material behaves under a physiological environment. The degradation profile reflects the length of time a scaffold would support the newly grown tissues, vascularization, and mechanical support. The scaffolds act as an active extracellular matrix that allows cell attachment, migration, and differentiation. These materials are replaced with natural cells after regeneration. The ideal scaffold should degrade naturally to avoid immune reactions and complications after performing its function. The rate of degradation should complement the rate of regeneration. Therefore, a degradable material is desired to avoid second surgery and infections within the body. The hydrolytic degradation is linked to the mechanical strength of the scaffold as well as its chemical nature. Gelatin contains carbonyl and amide functionalities that are water-loving and prone to degradation. However, its blend with other polymers and the addition of fillers prevents the rapid degradation of the gelatin (Ghorbani et al., 2016). The degradation rate of PVA is slower than gelatin, and it is stable in the body’s physiological environment, making it a suitable candidate for bone tissue regeneration (Xu et al., 2020). The degradation of PVA occurs via hydrolytic cleavage of the polymeric backbone. The degradation rate complements well with the mechanical results.

**FIGURE 5:**
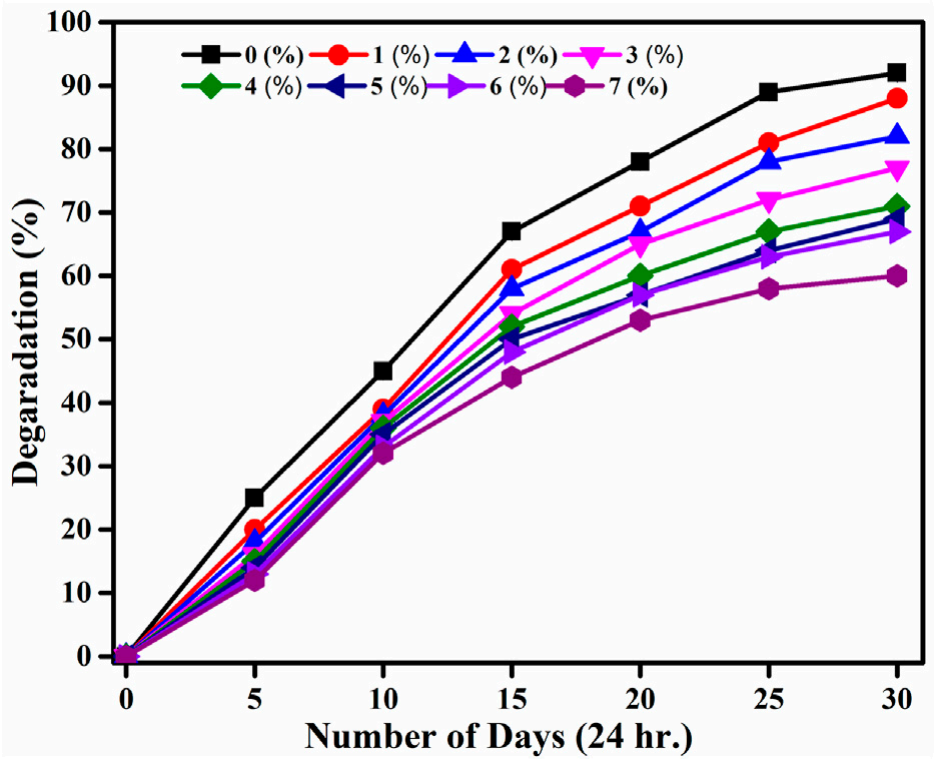
*In-Vitro* Degradation of PVA/Gelatin/WH Composites from 0 (black), 1 (red), 2 (blue), 3 (purple), 4 (olive green), 5 (royal blue), 6 (navy blue), and 7 (magenta) wt%.

The degradation studies of the PVA/Gelatin/WH composite show that all the compositions are stable for 30 days in the PBS solution, a time required for complete regeneration of bone tissue (Figure 5). However, the degradation rate of the pristine membrane is higher than the WH-containing membranes and is inversely related to the WH concentration. This could be due to polar functionalities of PVA and gelatin that are prone to degradation. However, after the addition of WH NPs, the degradation rate became slow, and the membrane with 7% WH NPs degraded up to 60% on the 30th day. The weight loss for the pristine membrane was 92% on the 30th day of the incubation in PBS medium. The weight change did not occur significantly in the first 10 days of the study, followed by a slight decrease in the weight of the polymeric composite. The reduction in weight loss after adding filler could be due to the formation of new noncovalent interactions between WH NPs and PVA/Gelatin chains that resulted in the formation of stable structures.

Moreover, WH NPs could have distributed themselves into the polar cavities of polymeric chains, resulting in an overall increase in the mechanical strength by loading 1 wt. %to 7 wt% of the WH and a decrease in the hydrolytic degradation, suggesting the stability of the prepared scaffold under the physiological environment (Chaudhuri et al., 2016). It was reported earlier that the degradation rate increases with the addition of filler which could be due to the higher solubility of the fill in the PBS, resulting in a drastic change in pH (Raafat and Abd-Allah, 2018). Present results show that the polymer matrix is not disrupted to release WH in the PBS medium. Therefore, no drastic change in pH was observed even after 25 days of incubation. The pH changed from 7.34 to 7.0 in the case of a 7 wt% loaded WH composite. Moreover, the dissolution of alkaline ions like Magnesium and Calcium exerts a buffering effect that compensates for the pH change because of the degradation of composite components. This buffering effect is beneficial as it prevents the inflammatory response that occurs because of the degradation of the polymer matrix (Raafat and Abd-Allah, 2018).

PVA and gelatin are hydrophilic, leading to higher uptake of the buffer solution that promotes hydrolytic degradation. However, the degradation rate is not fast, possibly due to lower buffer intake and stabilization kinetics with time. In the present case, the degradation of WH containing polymeric blend was slower than the pristine blend, which could be due to the formation of noncovalent bonds of the polymer side chains with the WH or due to the presence of WH within the polymer matrices that prevented the accumulation of water within the empty spaces (Satpathy et al., 2019). Due to the extra stability of the WH in comparison to HA, our prepared composite has demonstrated a degradation period that complements well with the healing time. Moreover, the presence of filler in our composite restricted the water uptake or peptide bond formation thereby reducing weight loss (Bakravi et al., 2018) Figure 6.

**FIGURE 6:**
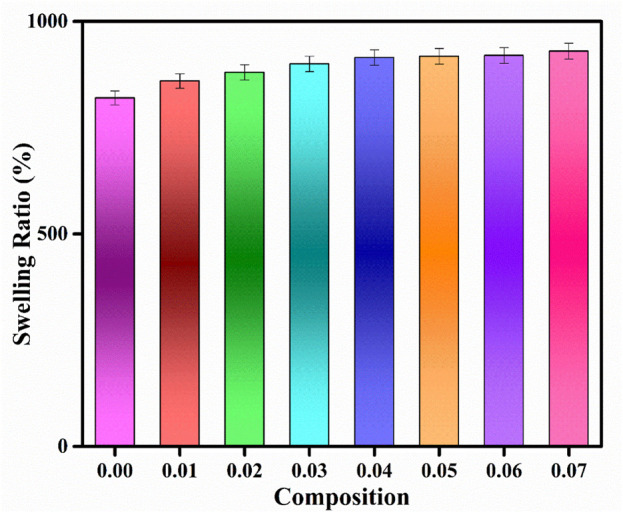
Swelling behavior of PVA/gelatin/WH composite.

### 4.5 Swelling ratio of PVA/Gelatin/WH composites

The hydrophilicity and swelling ratio are very important for bone tissue regeneration applications. A hydrophilic surface improves cell viability and proliferation. The swelling ratio in the PVA-PVP-HA blends has been reported to increase to 350% because of the hydrophilic nature of PVA and HA (Chaudhuri et al., 2016). The same could go with WH since it has $\text{HP}O_{4}^{2-}$ the group just like HA. It has a negative surface charge that helps bind polar groups on the surface and forms noncovalent linkages with polymers during scaffold formation. Gelatin also improves the water uptake ability of the scaffold because it has carboxylic and amine functional groups that are polar and hydrophilic (Ghorbani et al., 2016). The water uptake ability of HA/PVA/Gelatin is up to 300%. The porosity of the polymeric scaffold and surface roughness also play an essential role in the water uptake ability of the blend, along with its polar nature. The free spaces in the polymer matrix can hold endosomes’ water. Moreover, since PBS is a buffer, leaching the active ions from the scaffold decreases osmotic pressure (Wang et al., 2008). In the present investigation, the swelling ratio in the case of the pristine membrane was above 800%, while with an increase in the WH concentration, it increased linearly up to 930%. The membrane was flexible after 24 h, suggesting good mouldability under the body’s physiological environment (Anugrah et al., 2020). Polymeric composites have soft and wet forms that mimic the extracellular matrix of the human body and thus improve biocompatibility (Głab et al., 2021). This phenomenon suggests that the composite could establish a stable swelling ratio under the physiological environment (Nagahama et al., 2009). Excellent hydrophilicity of the composite increases cell affinity (Farag and Yun, 2014). The scaffolds should have an exceptional swelling ability when subjected to humid environments. Swelling is an imperative feature for scaffolds because the scaffold’s ability to store and conduct water through the matrix is essential to attain proper cell signaling and nutrition. The polymer chains should soak water through reversible interactions with water molecules through hydrogen bonds. The hydroxyl group of PVA and the polar nature of gelatin and WH improve the water uptake ability of the composite material. The PVA chains entangled with gelatin during the scaffold preparation because of the noncovalent interactions. These noncovalent interactions are responsible for the reversible exchange of water molecules with the polymer (Pineda-Castillo et al., 2018). The scaffolds possessing good swelling have a high surface area to volume ratio that improves the probability of cell growth by facilitating the entrance of oxygen and nutrients to the composite scaffold. However, the swelling must not be too high because the degradation rate increases and mechanical strength decreases at a higher value (Batool et al., 2020).

In our study, no significant increase in the swelling ratio with an increase in the concentration was observed which could be due to a negative surface charge that has made noncovalent linkages with the polymeric chains and reduced the availability of polar groups for water absorption. The uncontrolled swelling would result in unchecked degradation that is not required for bone tissue regeneration (Raafat and Abd-Allah, 2018).

### 4.6 Surface roughness of PVA/Gelatin/WH composites

**FIGURE 7:**
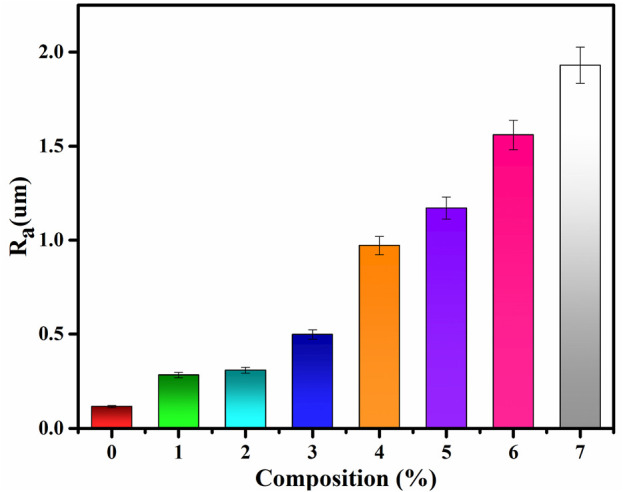
Surface roughness of PVA/gelatin/WH composite.

An increase in the surface roughness helps good cell adhesion on the scaffold surfaces. The surface roughness is increased by adding filler to the polymer matrix (Jose et al., 2020; Xu et al., 2020). In our study, the surface roughness increased linearly with an increase in the concertation of the filler (Figure 7). The Increase in the surface roughness can also be observed in the SEM images above.

### 4.7 Surface wettability of PVA/Gelatin/WH composites

**FIGURE 8:**
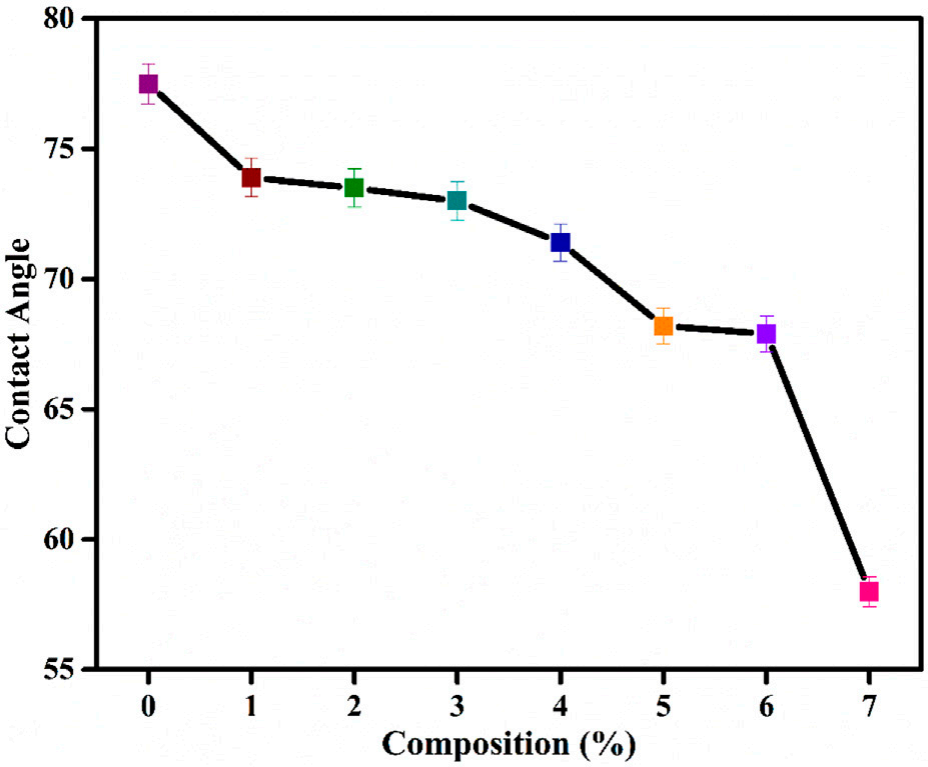
Surface wettability of PVA/Gelatin/WH Composite.

The chemical composition and topography of the membrane surface play a critical role in wettability. Gelatin and PVA are hydrophilic, resulting in good wettability (Kenawy et al., 2019). The lower contact angle suggests a good affinity of the composite for water (Tedesco et al., 2016). The average contact angle for PVA is 18.9^°^ (Satpathy et al., 2019). The surface wettability data of PVA/Gelatin/WH membranes confirm that all surfaces are hydrophilic (Figure 8), suggesting their suitability for cell proliferation and growth. There is a decrease in the contact angle with an increase in the WH NPs concentration, where 7 wt% composite showed the best wettability among all the samples.

## 5 Conclusion

WH NPs reinforced PVA/Gelatin matrix has been prepared using a simple solvent casting technique. The ATR-FTIR results of the PVA/Gelatin/WH composite confirm the formation of noncovalent interactions between WH NPs and PVA/Gelatin chains. The SEM analysis confirms an increase in the surface roughness with an increase in the WH NPs. The optical profilometry results also complement SEM results. The maximum tensile strength obtained is at 7 wt% loading of the WH, which is far higher than the PCL/WH composite and 153% more than the pristine polymer blend. Similarly, strain increased linearly with the addition of WH up to 7 wt% loading, and then a gradual decrease in the pressure was observed to 10 wt% loading. The increase in strain was 179% compared with the pristine blend. The prepared composite has good surface wettability and swelling properties and is biodegradable. Although the degradation rate is slow for our composite, it complements the body’s healing rate well. These results demonstrate that the prepared composite has all the properties required for a scaffold to be used for bone tissue regeneration. However, a detailed *in vivo* study may shed further light on the suitability of the composite for biomedical applications as scaffolds.

## Data Availability

The original contributions presented in the study are included in the article/Supplementary material, further inquiries can be directed to the corresponding author.
